# Bouveret Syndrome: A Rare Form of Gallstone Ileus

**DOI:** 10.7759/cureus.14042

**Published:** 2021-03-22

**Authors:** Farhan A Shah, S. M Winkle, Tyler Truitt, Gilad Guez, Kevin Draper

**Affiliations:** 1 Internal Medicine, Lewis Gale Medical Center, Salem, USA

**Keywords:** bouveret's syndrome, gallstone ileus

## Abstract

Bouveret’s syndrome is a rare variant of gallstone ileus characterized by a gastric outlet obstruction due to the impaction of a gallstone lodged in the duodenum, resulting from a cholecystoduodenal fistula. It accounts for only one to three percent of cases of gallstone ileus. We examine a case of Bouveret syndrome in an elderly Japanese female who presented with vomiting and decreased oral intake. Subsequent imaging found a gallstone ileus due to a bilioduodenal fistula. She underwent exploratory laparotomy enterolithotomy which found a large black gallstone located in the small bowel and confirmed the presence of the fistula. Despite its relative rarity, Bouveret syndrome carries a high risk of morbidity and mortality.

## Introduction

Bouveret’s syndrome is a rare variant of gallstone ileus. The syndrome is defined as a gastric outlet obstruction secondary to impaction of a gallstone lodged in the duodenum, caused by migration through the formation of a cholecystoduodenal fistula [1]. We present a rare case of Bouveret syndrome presenting as a gallstone ileus secondary to the formation of a cholecystoduodenal fistula.

## Case presentation

A Japanese female nonagenarian with a past medical history of non-insulin-dependent diabetes mellitus presented to the emergency department complaining of nausea and vomiting. She reported at least three daily episodes of emesis consisting of nonbloody, nonbilious, partially digested food for two days. She also endorsed dysphagia to solids and fluids with decreased oral intake for several days. Vital signs showed that she was afebrile, normotensive, with regular rate and rhythm. On physical examination, the patient had mild epigastric tenderness along with nausea that worsened with palpation. Initial labs were significant for a blood urea nitrogen (BUN) of 42 mg/dL and a creatinine of 2.2 mg/dL. She was managed conservatively with intravenous fluids and antiemetics for what was initially assumed as a case of gastroenteritis.

**Figure 1:**
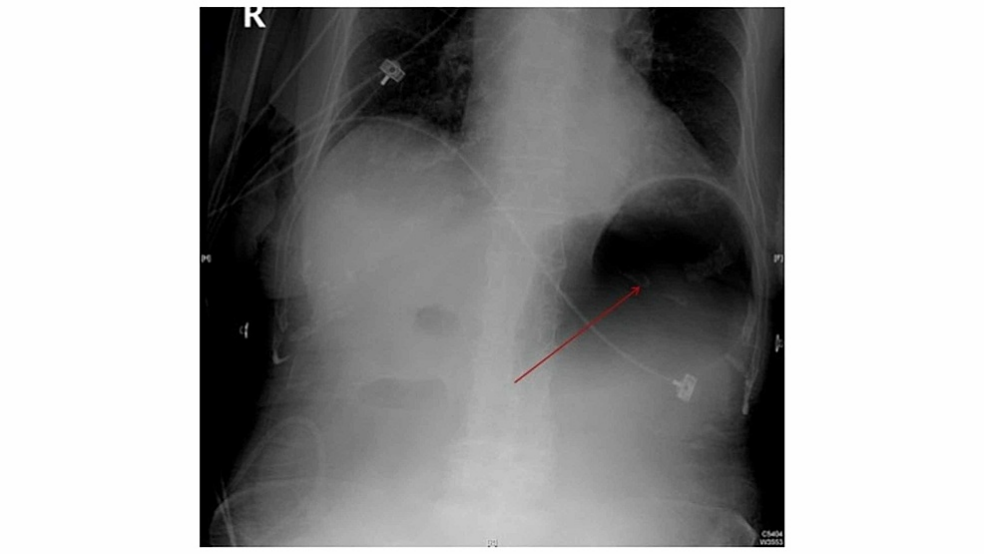
Abdominal radiograph revealed a distended stomach (red arrow) concerning for gastric outlet obstruction

**Figure 2:**
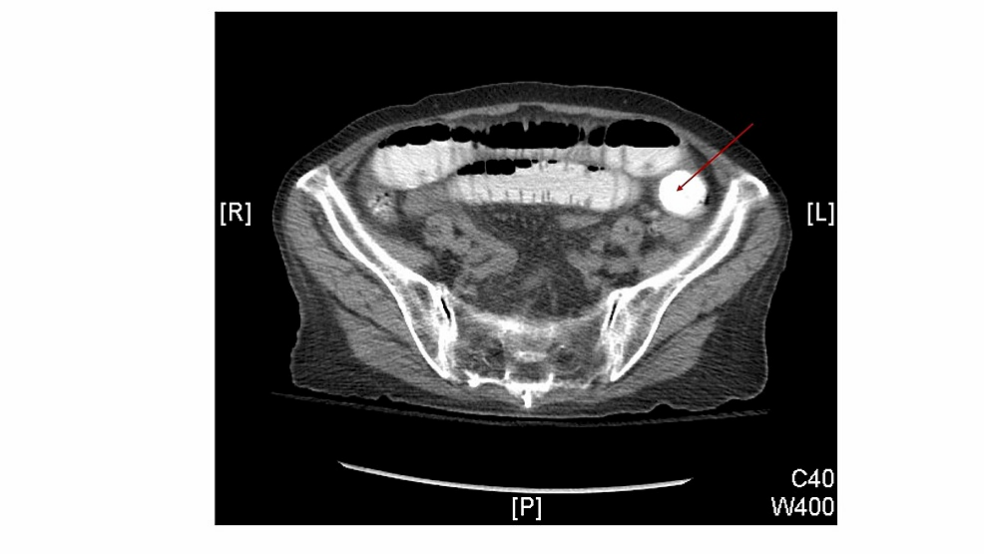
CT scan of the abdomen and pelvis with contrast revealed blockage of the left lower quadrant consistent with gallstone ileus (red arrow) due to a bilioduodenal fistula

**Figure 3:**
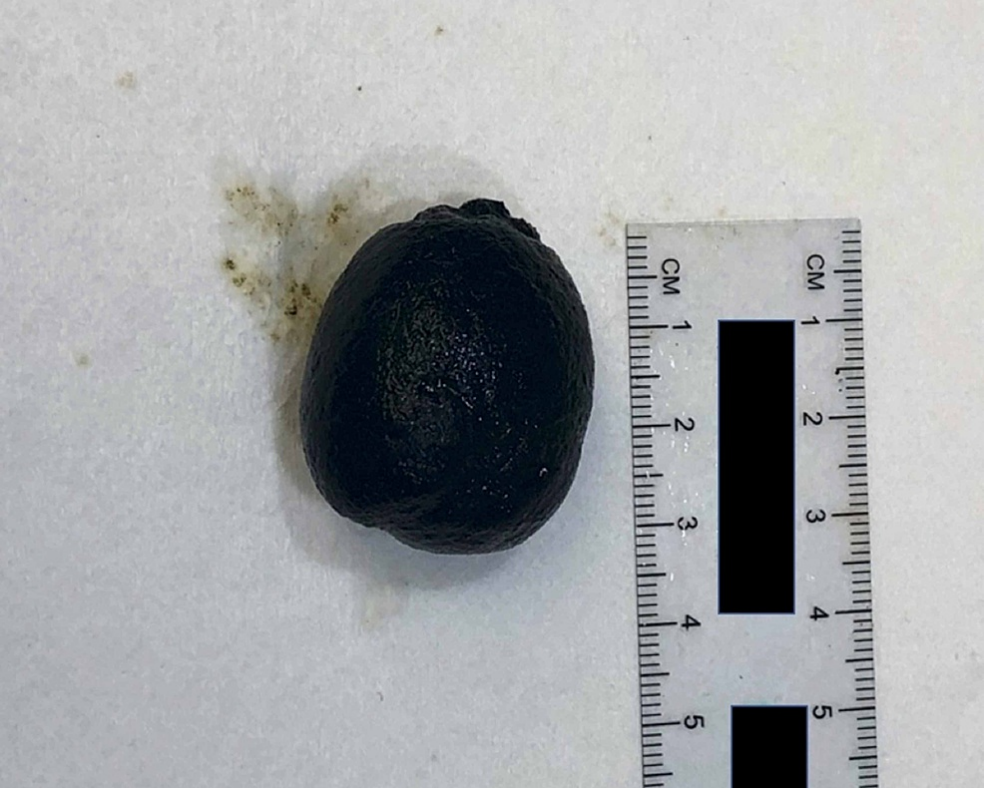
The gallstone was approximately 3.5 cm and black in color

The patient complained of abdominal pain and distention the following day. Abdominal radiograph revealed a distended stomach concerning for gastric outlet obstruction (Figure 1). A subsequent small bowel follow-through series further supported these findings. A CT scan of the abdomen and pelvis with contrast revealed blockage of the left lower quadrant consistent with gallstone ileus due to a bilioduodenal fistula (Figure 2). The patient’s bowel distention and intolerance of oral intake progressed. General surgery was consulted and they successfully performed an exploratory laparotomy enterolithotomy. Intraoperative findings confirmed a large gallstone in the small bowel secondary to a bilioduodenal fistula. The gallstone was approximately 3.5 cm and black in color (Figure 3). Upon further review of previous imaging, it was discovered that the patient had the same large gallstone present in her gallbladder one month prior to her presentation. These findings indicated that the gallstone had travelled through the newly formed cholecystoduodenal fistula and lodged in the left lower quadrant of the abdomen.

Postoperatively, the patient did not tolerate nasogastric tube clamping due to nausea and vomiting. Repeat CT scan confirmed small bowel obstruction and a perforated duodenal ulcer. The patient underwent a second exploratory laparotomy to repair the perforation, leading to a small bowel resection with admission to the intensive care unit. After transfer, the patient had persistent poor appetite and decreased oral intake. Esophagoduodenoscopy was performed for placement of a percutaneous endoscopic gastrostomy tube. During the procedure, a 14 mm fistula was noted at the junction of the duodenal bulb and the second part of the duodenum, consistent with the patient’s suspected cholecystoduodenal fistula. The patient appropriately recovered from the procedure, was able to tolerate oral intake, and was stable for discharge.

## Discussion

Bouveret syndrome was first classified in 1896 by French physician Leon Bouveret and was described as a form of gastric outlet obstruction due to an impacted gallstone in the duodenal bulb after traversing through a choledochoduodenal fistula [1]. Bouveret syndrome is an extremely rare variant of gallstone ileus, accounting for only one to three percent of cases of gallstone ileus [1]. Elderly women have been found to be more commonly affected, the median age being 74 years old. Risk factors include gallstones greater than two centimeters in size and elderly age. Mortality can range from 12-20% with a strong associated risk of severe ascending cholangitis [1,2]. In general, gallstone ileus causes one to four percent of all cases with small-bowel obstruction, with the most prevalent region of impaction located in the distal ileus [3,4]. A proximal obstruction, such as in the duodenum, is uncommon and accounts for one to three percent of all cases [3]. Generally, gallstone ileus occurs in 0.3-0.5% of patients with cholelithiasis [5].

Cholelithiasis is one of the most common diseases of the intestinal tract, with a reported presence of up to 15% based on one autopsy study [6]. The risk factors contributing to cholelithiasis are many and include diet, age, gender, BMI, and ethnicity, with higher prevalence in Latin American and Native American populations. Additionally, many medical conditions can contribute to the development of gallstones, including pregnancy, non-high-density lipoprotein (HDL) hyperlipidemia, Crohn’s disease, as well as a variety of blood disorders [6]. 

Gallstones can be divided into two categories: cholesterol and pigment. Cholesterol stones are the most common and comprise nearly 80% of stones in the Western world [6]. Cholesterol stones are formed due to the supersaturation of the hydrophobic cholesterol and bile acids. Pigment stones can be further subdivided into brown and black stones. Black pigmented stones are formed due to supersaturation of unconjugated bilirubin and the formation of calcium bilirubinate [6]. Brown pigmented stones should be considered a separate entity and are formed secondary to other causes, most notably infection and bile stasis. Bacteria such as *Escherichia coli *secrete the enzyme β-glucuronidase, which acts on soluble conjugated bilirubin, leading to the formation of insoluble unconjugated bilirubin [7]. This process, in combination with calcium and gut microbiota, forms the brown stones. Other causes can include parasitic etiologies, such as infection with *Ascaris lumbricoides* (roundworm) or *Clonorchis sinensis* (liver fluke), causing bile stasis and stone formation [7].

It has been documented that impacted gallstones can range in size from 2-10 cm, with a mean size of 4.3 cm. Gallstones greater than five centimeters are more likely to become impacted, which may result in a complication known as gallstone ileus. This occurs when the wall of the gallbladder fistulizes with the gastrointestinal tract. The stone then passes through the fistualization and becomes impacted, creating an obstruction [5]. In the case of Bouveret syndrome, a cholecystoduodenal fistula is formed followed by one or more stones obstructing the duodenum at the point of the gastric outlet [7].

The diagnosis of Bouveret syndrome includes laboratory studies and imaging. Laboratory findings associated with Bouveret syndrome can include leukocytosis and an acute kidney injury, as seen in this patient [3]. If obstructive jaundice is suspected, depending on the level of gallstone impaction, an elevation in both total and direct bilirubin, gamma-glutamyl transferase (GGT) and alkaline phosphatase (ALP) can be seen [3]. While laboratory studies are helpful in diagnosis, various imaging modalities are more useful in identifying the underlying process. CT scan is the best-known option as it has high sensitivity and specificity and identifies the ectopic gallstone as well as its location. The abdominal radiograph can be used, but due to the extensive gas formation and as most gallstones are radiolucent, the radiograph is very limited in diagnosing the underlying cause of the gastric outlet obstruction. Rigler’s triad (pneumobilia, bowel obstruction, and ectopic gallstone visualized on abdominal radiographs) is very rare and two of the three are seen in less than 50% of cases [4]. Oral contrast increases the sensitivity of the modality but is often limited by the patient's inability to tolerate consumption of the contrast due to emesis. Magnetic resonance cholangiopancreatography (MRCP) is an effective alternative as there is no need for oral contrast and can differentiate between fluid and the gallstone while being able to identify the fistula as well [3]. Gas formation limits the usefulness of ultrasound in the diagnosis of Bouveret syndrome. Endoscopy can be diagnostic and therapeutic but may be unable to identify the stone due to mucosa overlying the embedded stone [4]. 

Treatments include conservative, endoscopic, and surgical therapies. A conservative approach may be favorable if the stone is likely to be non-obstructive (less than 2.0-2.5 cm) as patients with this syndrome tend to be geriatric with an associated higher risk for surgical complications. A conservative approach would allow the stone to pass further into the small bowel, decreasing the risk of complications by surgical intervention but possibly resulting in distal obstruction [8]. Stone extraction by endoscopic lithotomy should be attempted as the first-line therapy and is favorable as well and will often not require repair of the cholecystoduodenal fistula. [1,9]. Due to its low success rate (less than 10%), failed endoscopic retrieval is often followed by lithotripsy or surgical means [1]. Lithotripsy may be considered in an attempt to fragment the gallstone by several methods. If endoscopic mechanical lithotripsy is unsuccessful, extracorporeal shock wave lithotripsy (ESWL) or laser lithotripsy have been utilized [10]. However, the resulting stone fragmentation by lithotripsy might also lead to fragment migration and consequently, a distal obstruction in the terminal ileum [1]. While surgical management remains the mainstay of treatment following the failure of less invasive methods to relieve the obstruction, there are no favored operative strategies discussed in the current literature [7,11]. Laparoscopic technique is preferred to open surgery, as open surgical management is associated with increased morbidity and mortality. Though no particular surgical approach is agreed upon and is often patient-specific, laparoscopic enterotomy or duodenotomy should be considered if expertise and patient stability permits [11].

## Conclusions

Despite its relative rarity, Bouveret syndrome carries a high risk of morbidity and mortality and clinicians should remain cognizant of its presentation and offer prompt treatment accordingly. Further documentation of similar case reports will lead to improved surgical protocols and planning for this syndrome, along with a greater awareness of the disease presentation.
